# Bio-mediated detoxification of heavy metal contaminated soil and phytotoxicity reduction using novel strain of *Brevundimonas vancanneytii* SMA3

**DOI:** 10.1016/j.heliyon.2023.e22344

**Published:** 2023-11-17

**Authors:** Ankita Ghosh, Diksha Sah, Moumita Chakraborty, J.P.N. Rai

**Affiliations:** Department of Environmental Sciences, Govind Ballabh Pant University of Agriculture & Technology, Pantnagar, 263145, Uttarakhand, India

**Keywords:** Heavy metals, Biosorption, Soil contamination, *Brevundimonas* sp.

## Abstract

Heavy metals pose a serious environmental threat on a global scale due to their toxicity towards livings. Therefore, removing harmful metals from the environment has become more challenging in recent years. The objective of this study is to isolate, examine, and characterize naturally existing bacteria that possess the ability to mitigate and detoxify heavy metals such as cadmium, mercury, and lead. The selected bacteria SMA3 actively demonstrated metal tolerance during screening and was then employed for biosorption study using a lab-scale technique. The bacterium belonged to *Brevundimonas* sp., according to 16 S rRNA analysis. To enhance the removal efficiency of SMA3, response surface methodology (RSM) was employed, resulting in the identification of optimized conditions (pH 7, temperature 30 °C and shaking speed 120 rpm) for achieving maximum removal percentage (69.5 % of Cd, 58.6 % of Hg, and 85.1 % of Pb) within 72 h. The structural changes induced by microbial treatment were demonstrated by comparing the findings of FESEM images and FTIR spectra confirming the disappearance of C ^ C, C]O peaks along with C]O, *C*–*O*–C, *C*–H, and O–H bond destabilization following bioaccumulation. Moreover, in terms of phytotoxicity evaluation, it was observed that the treated soil, containing both heavy metals and the selected potent bacterial strain, exhibited reduced toxicity, resulting in improved germination and growth parameters for the seeds of *Solanum lycopersicum* (tomato plant). Overall, the selected bacterial strain demonstrated its potential for effectively removing multiple metals from the metal contaminated environment.

## 1 Introduction

Rapid industrialization has facilitated product availability in recent years at the expense of growing environmental pollution owing to inadequate infrastructure for waste disposal [1]. Mining and industrial activities are essential for economic progress, especially in developing nations [2]. These activities, however, have resulted in the broad dispersion of heavy metals in the various components of ecosystem, and has caused numerous forms of health problem [3,4]. Heavy metals are naturally occurring elements, denser than water (5 g cm^−3^) and have an atomic number greater than 20 [5,6]. Heavy metals, such as Cu^2+^, Zn^2+^, Fe^2+^/Fe^3+^ etc., are necessary for many crucial biological processes (such as the metabolism of proteins, nucleic acids, carbohydrates, and lipids) in human physiology. However, even at somewhat larger doses, toxicological symptoms start to appear [7]. Heavy metals are regarded as potentially harmful pollutants due to their inability to degrade or be destroyed [8], which increases the concentration of metal ions along the food chain and can result in significant environment and health problems [9,10]. Heavy metals can be anthropogenically or naturally mobilized within the environment, where they combine with organic molecules to produce a variety of hazardous chemicals [8]. Contrary to organic pollutants, chemical or biological processes cannot break down heavy metals into simpler forms, they can only be transformed into less hazardous species, for example, As^3+^ can be transformed into the less toxic form As^5+^, Cr^6+^ to Cr^3+^ and Hg^2+^ to Hg^0^ [[11], [12], [13]].

Numerous adverse outcomes emerge when the intake of heavy metals, such as Cd, Pb, As, and Hg, surpasses the recommended biological threshold [14]. Importantly, toxic metals do not have recognized physiological functions in the human body. Excessive quantities of these metals can pose harm by disrupting reproduction, biotransformation, and growth in various organisms, including humans [15]. Certain toxic metals, such as Cd, Hg, and Pb, are pervasive in industrial processes and constitute significant environmental pollutants [16]. The risks associated with these metals are well-documented; for instance, lead exposure can lead to intellectual impairments in children. When Cd, Hg, or Pb levels surpass permissible limits, they can induce organ damage, including nephrotoxicity, skin toxicity, neurotoxicity, hepatotoxicity, and cardiovascular toxicity, as these toxic metals may stimulate the generation of reactive oxygen species (ROS) [17,18].

In this context, bioremediation is regarded to be an essential approach for heavy metal removal from the environment, where living component act as a solar-powered pump capable of extracting harmful elements from polluted soil [19,20]. The bioremediation procedure often makes use of microorganisms or their enzymes to degrade and subsequently detoxify hazardous substances in the environment [21]. Microorganisms have developed diverse methods for living in heavy metal contaminated environments, such as valence transformation, extracellular chemical precipitation, volatilization [22], biosorption, bioaccumulation, biotransformation and biomineralization [23]. Moreover, bioremediation encompasses all procedures and actions used to restore a contaminated environment to its pre-contamination state [24,25]. This approach holds promise for addressing various environmental challenges, including the elimination of xenobiotic compounds in agrochemical and petrochemical sectors, remediation of oil spills, treatment of heavy metal pollution in sewage, sludge, and marine sediments, along with other related issues [26].

To limit industrial exploitation of chemicals and to attain pollution-free environments, microbial bioremediation of heavy metals is an efficient, cost-effective, and environmentally benign approach [27]. Microorganisms have been assessed for detoxifying metals in various systems, however, the effectiveness is constrained by cell viability, metabolic activity, and enzymatic efficiency [28]. Therefore, the primary objective of this study is to investigate the capacity of naturally existing bacteria to mitigate and detoxify heavy metals (in particular Cd, Hg and Pb) while characterizing and identifying the bacterium at the species level. In order to achieve optimal physicochemical conditions, a statistical evaluation was conducted, and the RSM were employed to determine the influence of variables on the removal efficiency of heavy metals. The surface elemental profiling of the bacterial biomass following bioaccumulation was performed using the FESEM technique. Additionally, chemical analysis of the heavy metals was carried out using analytical techniques, including UV–Visible spectroscopy and FTIR measurements. These analyses provided valuable insights into the chemical changes that occurred during the removal of heavy metals. Furthermore, the phytotoxicity assessment on *Solanum lycopersicum* (tomato plant) was studied to evaluate the impact of the treated soil on plant growth and health.

## 2 Materials and methods

### 2.1 Soil sample collection and heavy metal analysis

Soil samples contaminated with various heavy metals were collected randomly from disposal sites of an Indian ink and chemical industry located at SIIDCUL (28.9787° N, 79.3851° E), Pantnagar, Uttarakhand, India. The sampling was done between 5 and 15 cm beneath the topsoil using tools like spade, trowel, and shovel. To eliminate any stones, concrete, bulky residues, or other pollutants, the soil samples were air-dried, ground, and passed through a 2 mm mesh size. The processed soil samples were then stored in sterile sampling bags at 4 °C for further analysis [29].

Samples were processed for determination of Cd, Hg, and Pb using an atomic absorption spectrophotometer (AAS). However, the analysis of Hg using AAS yielded suboptimal sensitivity owing to mercury's notable volatility and significant vapor pressure at room temperature [30]. To tackle this issue, cold vapor atomic absorption spectrometry (CVAAS) was employed for Hg analysis. Heavy metals were extracted from the soil using the acid digestion technique. In this process, a 3:1 solution of hydrochloric and nitric acids in 24 ml of aqua regia was used to treat 1.5 g of dry soil. The acid-treated soil was then heated on a hot plate at 100 °C for about 2 h, until the solution became translucent. After cooling and filtration, the digested soil contents were diluted with double distilled water to make a 50 ml solution. Subsequently, this diluted filtrate was subjected to analysis for the presence of Cd and Pb using AAS, while Hg was determined using CVAAS [30,31].

### 2.2 Heavy metal stock solution and media preparation

The metal stock solutions were prepared separately using cadmium chloride (CdCl_2_), mercuric chloride (HgCl_2_) and lead acetate (Pb(C_2_H_3_O_2_)_2_), for Cd, Hg and Pb, respectively at 1000 ppm. The metal solutions were thoroughly mixed to ensure that the salts were completely dissolved [32]. Working solutions of various concentrations were diluted from the stock solution as required for the experiment. For bacterial cultivation and isolation, Luria Bertani (LB) agar and LB broth medium were used [31]. LB agar is a culture medium made up of sodium chloride (0.5 g/L), tryptone (10 g/L), yeast extract (5 g/L) and agar (15 g/L). The primary distinction between LB broth and LB agar lies in the absence of agar in LB broth.

### 2.3 Isolation of heavy metal resistant bacteria

Effective implementation of the enrichment culturing technique is pivotal in isolating microorganisms capable of pollutant degradation while excluding non-performers. This ensures the growth of an active and high-density population of pollutant-degrading microorganisms [33]. In the present work, bacterial strains were isolated from the soil sample using a two-step process involving enrichment and serial dilution. To enrich the soil, 50 ppm of Cd, Hg, and Pb were added to a 90 ml LB broth medium, maintaining the pH at 7.0 ± 0.2. The enrichment was carried out aerobically for 3 days at 30 °C with constant shaking. After enrichment, 1 ml of the sample was taken and subjected to serial dilution with distilled water. The diluted suspensions were then spread on LB agar plates, and colonies with distinct morphological traits were carefully selected and purified. For purification, a total of 36 distinct bacterial isolates were selected.

### 2.4 Screening and determination of MIC of heavy metal resistant bacteria

The initial step in the selection process involved subjecting the selected bacterial isolates to a screening procedure. This screening procedure included measuring their optical density at 600 nm (OD600) and determining their maximum absorbance (*λ*_max_) using a UV–vis spectrophotometer. The screening was conducted by introducing 50 ppm of Cd, Hg, and Pb into LB medium containing the selected bacterial isolates. Measurements were taken consistently at a temperature of 30 °C, with intervals of 24 h being maintained over a 5-day period. Following screening, the top 10 bacterial isolates displaying the highest OD600 values, signifying strong resistance to heavy metals, were selected for further analysis.

Minimum inhibitory concentration (MIC) was defined as the concentration at which the bacterial isolates ceased to proliferate, signifying their threshold of heavy metal tolerance [32]. To determine the MIC of these 10 selected isolates, each of them was independently grown on LB agar plates enriched with an initial concentration of 50 ppm of Cd, Hg, and Pb at 30 °C for 24–48 h. The bacterial isolates were transferred onto fresh LB agar plates, and the metal concentration was gradually elevated until the isolate could no longer grow. The experiment was conducted separately for each metal. After assessing the MIC values, the bacterial culture with the highest MIC for Cd, Hg and Pb, denoted as SMA3, was identified and selected for further investigations.

### 2.5 Identification of the selected bacteria SMA3

The bacterial strain SMA3 was cultured on LB agar plates and incubated at 30 °C for 24 h for characterization. To gain insights into its characteristics, various parameters were evaluated to assess colony morphology including color, surface, margin, gram staining, and elevation. Additionally, standard biochemical tests, such as catalase and oxidase tests; gelatin, casein, and starch hydrolysis; citrate utilization; nitrate reduction and urease production, were performed according to established protocols [34]. Furthermore, IMViC tests were conducted using test kits from Hi-Media to further explore the properties of the strain SMA3.

### 2.6 16 S rRNA amplification and sequencing

The identification of the selected strain was accomplished through the analysis of its 16 S rRNA gene sequences. Subsequently, the DNA from the bacterial culture was isolated and its quality was evaluated using a 1.0 % agarose gel. The gel analysis revealed the presence of a singular high-molecular weight DNA band. The universal primers 27 F (5′—AGA GTT TGA TCC TGG CTC AG—3′) and 1492 R (5′—TAC GGG TAC CTT GTT ACG ACT T—3′) were used to amplify the 16 S region [35]. Following PCR amplification, the samples underwent a quality check using 2 % agarose gel electrophoresis. Sample that passed quality control were proceeded for purification using QIAGEN QIAquick PCR Purification Kit (cat. No. 28104) and purified samples were taken for sequencing. Using the Big Dye™ Terminator V3.1 kit, a sequencing PCR reaction was set up in an Applied Biosystems MiniAmp™ Plus Thermal cycler. Using aligner software, the 16 S rRNA gene consensus sequence was produced from forward and reverse sequencing data. The NCBI GenBank ‘nr’ database was searched using the 16 S rRNA gene sequence. The first 10 sequences were chosen based on the maximum identity score and aligned using the multiple alignment application Clustal W [36]. Phylogenetic analysis and tree construction was carried out using the Weighbor programme.

### 2.7 Antibiogram pattern of the selected isolate

The antibiotic sensitivity of the selected bacterial strain was assessed using the disc diffusion method, also known as Kirby-Bauer method, with slight modifications [37]. Initially, the isolated strain was cultured in LB broth and incubated for three days at 30 °C to achieve sufficient growth. After incubation, 100 μl of the bacterial culture was spread evenly on freshly prepared LB agar plates using a sterilized glass spreader. Antibiotic discs containing different antibiotics, including ampicillin, cefuroxime, cephadroxil, doxycycline, penicillin, oxacillin, streptomycin, and tetracycline, were then placed equidistantly on the surface of the LB agar plates. Subsequently, the plates were incubated at 30 °C for 24–48 h, allowing the bacteria to interact with the antibiotics. After incubation, the plates were examined for the presence or absence of a zone of inhibition surrounding each antibiotic disc. A clear zone of inhibition indicates that the bacterial strain is sensitive to the antibiotic, while the absence of an inhibitory zone suggests that the strain is tolerant or resistant to the antibiotic [38].

### 2.8 Optimization of culture condition for heavy metal removal using RSM

The optimization of culture conditions for the removal of heavy metals (Cd, Hg, and Pb) was conducted utilizing a response surface methodology (RSM) approach, specifically employing the Box-Behnken Design (BBD) matrix. The study encompassed a total of 17 experiments, including three replicates at the central point. In this experiment, three critical factors and their respective ideal ranges were identified for heavy metal removal: pH (5, 6, 7, 8 and 9), temperature (10, 20, 30, 40 and 50 °C), and agitation speed (40, 80, 120, 160 and 200 rpm). These experiments were designed based on this matrix to assess both the individual and cumulative impacts of these chosen factors on the dependent variable, which, in this case, is the efficiency of heavy metal removal. The dependent variable under consideration was the removal of Cd, Hg, and Pb at initial concentration of 50 ppm in 100 ml of LB medium on 3rd day. The experiment was conducted separately for each metal. A polynomial equation of the second order (Eq. (1)) was developed using statistical analysis software (SAS) version 10.0, to analyze the interaction between the dependent and independent factors [39].

(1) $$Y=A_{0}+\sum_{j=1}^{k}A_{j}X_{j}+\sum_{j=1}^{k}A_{ii}X_{j}^{2}+\sum_{x-1}^{j=1}\sum_{j=2}^{k}A_{ij}X_{i}X_{j}+\in$$

where,

*Y* = anticipated-response (dependent-variable), *A*_*0*_ = constant, *A*_*ij*_ = interaction coefficient, *A*_*ii*_ = squared-coefficient, *X*_*i*_ and *X*_*j*_ are the proportions of independent variables and Ɛ is error. They indicate the response's linear, quadratic, and cross-product effects of the X_1_, X_2_, and X_3_ components, accordingly.

The formula, expressed as a function of coded variables, allows for predicting the response for specific levels of each component. The high values of the factors are denoted as +1, while their low levels are represented as −1 in the coded formula. By analyzing the factor coefficients, one can assess the relative significance of each element.

The experimental design, involved conducting seventeen experiments in the laboratory are given by the chosen matrix BBD. After a period of 3 days, 3 ml samples were extracted from each flask to determine the removal percentage of Cd, Hg and Pb. The optimization process was carried out based on the results obtained during these experiments [39].

### 2.9 Bioaccumulation study of Cd, Hg and Pb by SMA3

The bacterial isolate was cultivated in LB medium under optimal conditions of pH, temperature, and agitation rate. The objective of the study was to evaluate the tolerance of cultured bacteria to varying levels of cadmium (100, 200, 300, 400 and 500 ppm), mercury (50, 100, 150, 200 and 250 ppm), and lead (100, 300, 500, 700 and 900 ppm). The experiment was conducted separately for each metal. For the inoculation process, 4 ml of a previously grown bacterial culture was added to the enriched broth. The cultures were subsequently placed in an incubator for durations of 24, 48, and 72 h. At specific intervals, samples were retrieved from the flasks to assess the remaining concentration of the substrate. Following the incubation period, the broth underwent a centrifugation process lasting 20 min at 4000 rpm. A 2 mL portion of the resulting cell-free supernatant was collected at regular intervals for the assessment of heavy metals in the culture medium. Prior to determination of the heavy metal, both the pellet and the supernatant were individually treated with a mixture of acids (a combination of three parts hydrochloric acid and one-part nitric acid) to facilitate digestion [40]. AAS was utilized to calculate the residual quantities of Cd and Pb, whereas for residual Hg concentrations, CVAAS was employed. The efficiency of the microorganism to bioaccumulate heavy metal ions was then evaluated using the formula (Eq. 2) and is usually expressed with R (%) [41].

(2) $$R\left(\%\right)=\frac{\text{Initial}\;\text{heavy}\;\text{metal}\;\text{concentration}-\text{Residual}\;\text{heavy}\;\text{metal}\;\text{concentration}}{\text{Initial}\;\text{heavy}\;\text{metal}\;\text{concentration}}\times 100$$

### 2.10 Biostimulation for enhanced growth of selected isolate

A nutrient supplementation experiment, commonly referred to as biostimulation, was carried out to augment the growth of selected bacteria in LB broth. Carbon substrates, such as starch, glycerol, and glucose, were added, while for nitrogen sources, sodium nitrate (NaNO_3_), peptone, and urea were utilized. The experiment was conducted in 250 mL Erlenmeyer flasks containing 100 mL of LB broth. The broth was supplemented with 50 ppm each of Cd, Hg, and Pb, and 1 % (w/v) of each carbon and nitrogen substrate was added as mentioned earlier. The experiment was conducted individually for each substrate. To assess the growth of SMA3, the optical density at 600 nm (OD600) was measured after a period of 3 days. The OD600 measurement provided valuable insights into the bacterial growth and activity in the presence of different carbon and nitrogen substrates. This experiment aimed to identify the most suitable carbon and nitrogen sources that could accelerate the growth of SMA3 in LB broth and potentially enhance its ability to reduce heavy metals, making it more efficient for bioremediation applications.

### 2.11 Analytical analysis

Fourier Transform Infrared Spectroscopy (FTIR), Field emission scanning electron microscopy **(**FESEM) and Energy dispersive X-ray (EDX) was employed to elucidate the primary mechanisms through which the microorganism accumulates heavy metal ions.

The FTIR spectrophotometer was employed to evaluate the structural changes of the microorganism after contact with metal ions. In the control, LB broth medium was inoculated solely with the selected bacteria. Conversely, the experimental sample involved incubating the selected bacterial strain with the heavy metals (Cd, Hg and Pb). The mixture was then incubated at 30 °C for 3 days. After incubation, the broth underwent centrifugation at 4000 rpm for 20 min. Subsequent to centrifugation, the collected solid residue (bacterial biomass) underwent FTIR analysis to investigate the interaction between heavy metals and the functional groups present on the selected bacteria. By utilizing the control as a reference in KBr, a comparison was made between the analysis of the treated sample and its distinct peaks indicative of various bond stretching and bending motions [42]. The spectral wave numbers (cm^−1^) for the FTIR peaks ranged from 4000 to 400 cm^−1^.

Field emission scanning electron microscopy **(**FE-SEM) was used to examine the surface morphology of the bacterial samples, which revealed substantial differences in surface topography between the treated and control samples. The samples were purified using a phosphate buffer and ethanol gradient dehydration process, followed by drying under vacuum conditions. The bacterial samples were then gold polished and examined under a 10000× magnification FE- SEM. The presence of the additives was detected using an Energy dispersive X-ray (EDX) analyzer and FE-SEM. Its programming is designed to maximize data extraction from all specimens, including imaging and microanalysis, and has an electron backscatter diffraction attachment for analyzing sample texture [39]. For microscopic-level elemental distribution analysis, EDX patterns were acquired utilizing the Oxford EDX system in conjunction with a micro-analytical unit [43]. The regions investigated through EDX align in a linear fashion with the morphological inspection accomplished through FESEM. To mitigate charging effects, observations were conducted at an exceedingly low kV, assisted by a charge compensator. When utilizing EDX, samples were immediately checked with a FESEM and a 20.00 kV EHT accelerating voltage for the presence or absence of elements [44].

### 2.12 Phytotoxicity assay

The focus of this research was on investigating the effects of heavy metals and bioremediated soil on the growth and development of *Solanum lycopersicum* (tomato plant), during its seedling stage. The choice of the tomato plant for this study stemmed from its heightened sensitivity to pollutants, making it a valuable indicator in plant toxicity evaluations. To initiate the experiment, tomato seeds were sourced from the Horticulture Department at GBPUA&T, Pantnagar, Uttarakhand. Prior to their use, all seeds underwent a disinfection process involving a 10-min immersion in a 5 % sodium hypochlorite solution, followed by a thorough rinse with distilled water. This crucial procedure was carried out to ensure the cleanliness and preparedness of the seeds for subsequent testing [45].

For seed treatment, the seeds were soaked in 100 ml of a 48-h active culture of SMA3 in LB broth. They were incubated for 6 h to stimulate germination and overcome dormancy. Additionally, a control group was set up with seeds soaked in distilled water and left at room temperature for reference. After treatment, the seeds from each group were planted in pots and subjected to natural environmental conditions, including a temperature of 25 ± 2 °C and a 12-h light photoperiod. Each pot contained 15 seeds from each treatment, and the entire experiment was replicated three times to ensure accuracy.

Distinct treatment regimens were established for the experiment, encompassing various conditions. These included a control group featuring soil and distilled water (designated as T1), a group exposed to contaminated soil containing Cd, Hg, and Pb (referred to as T2), additional groups where the soil was treated with active SMA3 cultures alongside Cd (T3), Hg (T4), and Pb (T5). At the conclusion of a 45-day period, diverse growth parameters were assessed to gauge the effects. These parameters encompassed the germination rate, lengths of shoots and roots, fresh and dry weight of the plants, as well as the measurement of leaf area. Each treatment was subjected to these measurements to comprehensively evaluate their impact on the tomato plants. Seed germination percentage (GP) was quantified using Eq. (3), which was a part of the analysis process.

(3) $$\frac{\text{Total}\;\text{number}\;\text{of}\;\text{seeds}\;\text{germinated}}{\text{Total}\;\text{number}\;\text{of}\;\text{seeds}\;\text{sown}}\times 100$$

### 2.13 Statistical analysis

In this study, each experiment was performed with three replicates to ensure the reliability of the results. Excel 2021 was utilized to calculate trial errors, which were then represented as error bars in graphs or ± SD (standard deviation) in tables. The data from all the experiments, including the triplicate feedback, were collected and subjected to analysis using the analysis of variance (ANOVA) test in SPSS software. A confidence interval of 95 % was applied during the statistical analysis to assess the significance and reliability of the observed differences among the experimental groups.

## 3 Results and discussion

### 3.1 Heavy metal analysis and isolation of heavy metal resistant bacteria

The outcomes of heavy metal analysis unveiled the existence of Cd, Hg, and Pb within a concentration range of 0.746–0.971 ppm, 0.338–0.521 ppm, and 7.515–12.541 ppm, respectively. Remarkably, the concentration of Pb was found to be significantly higher when compared to the levels of other metals. Initially, a total of 36 isolated bacterial cultures were subjected to evaluation to assess their tolerance to heavy metals. Through optical density (OD) measurements, it was found that 10 bacterial isolates exhibited high absorbance values, indicating their superior ability to reduce heavy metals, making them promising candidates for further investigation.

### 3.2 MIC of the selected bacterial isolate

After identifying 10 bacterial strains with the high absorbance values in the initial screening, further testing was conducted to determine their capacity to withstand higher concentrations of Cd, Hg, and Pb. These selected bacterial isolates were cultivated on LB agar plates containing a range of metal concentrations. The experiments were conducted separately for each specific metal. Among all the bacterial isolates, the strain SMA3 demonstrated the highest resistance, as evidenced by its minimum inhibitory concentration (MIC) values against Cd, Hg, and Pb. The dose-dependent MIC values for SMA3 against Cd, Hg, and Pb revealed that Hg was found to be the most toxic, with a MIC of 650 ppm, followed by cadmium at 870 ppm, and lead at 1120 ppm **(S1)**. A comparison with other studies indicated that SMA3 showed a greater potential for heavy metal bioaccumulation compared to other bacteria. In a study by Nath et al. [46], different bacterial strains exhibited MIC values ranging from 50 to 600 ppm against Cu, Cd, Pb, and Zn. Similarly, Marzan et al. [31] observed that *Gemella* sp. displayed MIC values of 450 μg/mL for Cd, 360 μg/mL for Hg, and 900 μg/mL for Pb.

### 3.3 Characterization and identification of selected isolate SMA3

**Fig. 1:**
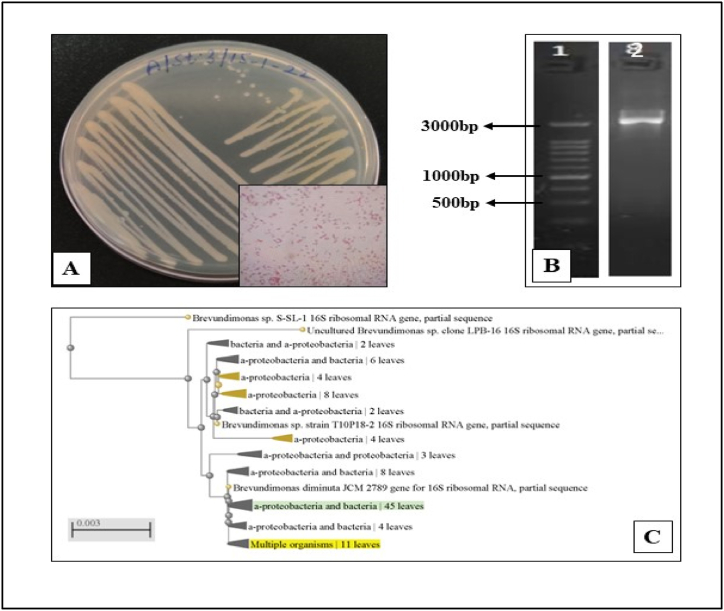
(A) Gram reaction demonstrated by bacterial isolate SMA3 **(B)** Analysis of the 16 S rRNA gene of SMA3 through agarose gel electrophoresis; Lane 1 - 1 kb DNA marker; Lane 2 - PCR product of SMA3 **(C)** Phylogenetic tree illustrating the relationship between *Brevundimonas vancanneytii* SMA3 and other closely related taxa, inferred from 16 S ribosomal RNA gene sequences.

The potent bacterial strain, SMA3, was identified based on a comprehensive analysis of its morphological, biochemical, and molecular characteristics. Morphological examination and gram staining revealed that the bacterium appeared as rod-shaped cells arranged in chains and identified as a gram-positive bacterium (Fig. 1A). In terms of appearance, SMA3 displayed a creamy white color with a smooth surface and irregular margins (Fig. 1A, Table 1). Biochemical characterization further confirmed its identity, showing that SMA3 exhibited positive reactions for nitrate reduction, citrate utilization, and urease production. However, it tested negative for methyl red, oxidase, and indole production. Carbohydrate utilization ability was also assessed for SMA3, revealing that it could actively utilize glucose, arabinose, rhamnose, and sucrose as carbon sources. However, it was unable to grow in the presence of adonitol, sorbitol, mannitol, and lactose **(**Table 1**)**.

**Table 1:** Morphological and biochemical characteristics of the selected strain SMA3.

Characteristics	Bacterial strain A3
**Morphological characteristics**	
Color	Creamy white
Surface	Smooth
Margin	Irregular
Gram staining	+ive
Elevation	Convex
**Biochemical characteristics**	
Catalase test	+
Oxidase test	–
Gelatin hydrolysis	+
Casein hydrolysis	–
Starch hydrolysis	–
Nitrate reduction	+
Urease production	+
Indole	–
Methyl red	–
Voges Proskauer	+
Citrate	+
**Carbohydrate utilization test**	
Glucose	+
Adonitol	–
Arabinose	+
Lactose	–
Sorbitol	–
Mannitol	–
Rhamnose	+
Sucrose	+

The 16 S rRNA gene of the selected bacterial isolate was fully sequenced, and its sequence was compared for similarities. According to the NCBI BLAST search software, the sequencing data showed a high identity (99–100 %) to *Brevundimonas vancanneytii*, with a bit score of 2617 and an E value of 1428. To further validate the phylogenetic relationship, a bootstrap test was performed, and the resulting tree displayed well-organized clusters of connected taxa. The tree was scaled, and branch lengths were calculated using the same units as the evolutionary distances used to construct the phylogenetic tree. Furthermore, genomic DNA was amplified, and the agarose gel electrophoresis picture revealed a 500 kb, 1000 kb, and 3000 kb 16 S rRNA gene fragment (Fig. 1B). The phylogenetic tree was constructed using the Weighbor programme (Fig. 1C).

### 3.4 Antibiotic tolerance and sensitivity

The selected bacterial isolate, identified as *Brevundimonas vancanneytii*, exhibited resistance to several antibiotics, including penicillin, tetracycline, doxycycline, ampicillin, cefuroxime, and oxacillin. However, it displayed sensitivity to streptomycin and cephadroxil (Table 2). Notably, several researchers have reported similar findings, highlighting that bacterial isolates with multiple metal resistance tend to exhibit robust resistance to specific classes of antibiotics [46]. Rasheed et al. [47] identified a correlation between the ability of bacterial species to withstand elevated concentrations of heavy metals and their resistance to antibiotics. In line with these observations, both *Pseudomonas* sp. and *Staphylococcus* sp. have been shown to demonstrate significant levels of antibiotic resistance as well as multi-metal resistance [46].

**Table 2:** Antibiotic resistance pattern of selected metal resistant isolate.

Antibiotic resistance	SMA3
Penicillin	+
Tetracycline	+
Streptomycin	–
Cephadroxil	–
Doxycyclin	+
Ampicillin	+
Cefuroxine	+
Oxacillin	+

### 3.5 Optimized condition for heavy metal removal using RSM

The Box-Behnken Design (BBD) matrix, serves as an experimental design technique for response surface methodology (RSM), has been extensively utilized to optimize operational parameters for the enhanced removal of heavy metals. In this study, a total of 17 test runs were conducted to determine the most effective combinations of temperature (A), pH (B), and shaking speed (C) that yield optimal results in the removal of heavy metals. The experiments were carried out using the specific configurations outlined in Table 3, with the outcomes of these trials being summarized in Table 4. The expressions representing the second-order polynomial equations for Cd (Eq. (4)), Hg (Eq. (5)), and Pb (Eq. (6)) are given as follows.

(4) +46.96+1.30*A+1.60*B+3.03*C–6.43*AB+2.52*AC+1.28*BC–11.27*A2–10.42*B2–11.72*C2

(5) +51.56+6.07*A–0.9000*B+2.45*C+0.3250*AB–0.1250*AC+0.0750*BC–14.17A2–10.17*B2–10.12*C2

(6) +70.50+8.24*A+0.6000*B+0.1125*C+4.85*AB+1.18*AC–4.45*BC–18.74A2–7.36*B2–9.24*C2

**Table 3:** Experimental range and levels of independent variables.

Factors	Coded levels		
	−1	0	+1
Temperature (°C)	10	30	50
pH	5	7	9
Shaking speed (rpm)	40	120	200

**Table 4:** Experimental values depicting the removal percentages of cadmium (Cd), mercury (Hg), and lead (Pb) utilizing the specific bacterial strain SMA3.

S. No	pH	Temperature (°C)	Shaking speed (rpm)	Cd removal (%)	Hg removal (%)	Pb removal (%)
1	9	30	40	35.3	19.6	51.4
2	7	50	200	36.2	31.3	50.5
3	9	30	200	31.6	29.6	50.2
4	7	30	120	47.4	52.5	72.6
5	5	50	120	20.9	31.3	33.5
6	7	10	200	39.6	25.5	61.3
7	9	10	120	32.9	32.1	45.6
8	7	30	120	49.6	49.7	75.4
9	7	30	120	53.7	50.3	68.7
10	5	30	200	19.5	23.3	31.3
11	5	10	120	21.6	15.3	38.9
12	7	30	120	56.4	40.9	65.4
13	7	30	120	50.7	41.4	70.4
14	7	10	40	26.5	20.9	48.4
15	5	30	40	22.7	23.4	37.2
16	9	50	120	33.5	22.4	59.6
17	7	50	40	22.8	21.6	55.4

It is possible to predict the response for particular levels of each element using the equation formulated in terms of coded variables. In this context, higher parameter values are represented as +1, while their lower values are indicated as −1. By examining the coefficients of these variables, the encoded equation can be utilized to determine the relative importance of the elements.

The accuracy of the resulting quadratic polynomial equation was assessed using the statistical F-test (ANOVA). The analysis of variance (ANOVA) for the constructed process model can be found in the **S2 A, B and C** for Cd, Hg and Pb, respectively. In the ANOVA, the degrees of freedom were nine for error and four for variance. The F-values of the model for Cd, Hg, and Pb were found to be 13.97, 8.93, and 15.10, respectively, indicating a significant model. The occurrence of such significantly high F-values due to noise is extremely rare. Probability values less than 0.05 (p < 0.05) indicated the significance of the model term. For Cd and Pb, terms AB, A^2^, B^2^, and C^2^ were identified as significant model terms, while for Hg, terms A, A^2^, B^2^, and C^2^ were significant, and the remaining interacting and non-interacting terms were considered non-significant. Model terms with p values exceeding 0.1000 were considered not significant. The p-values of 0.9734, 0.0452, and 0.1111 for Cd, Hg, and Pb, respectively, in terms of lack of fit, suggest that it is relatively minor compared to the actual error. An insignificant lack of fit is desirable for a well-fitting design.

To ensure the accurate anticipation of outcomes within the testing conditions, it is crucial to establish an appropriate model. The calculated coefficient of determination (R^2^) for the removal of Cd, Hg, and Pb was determined as 94.36 %, 92.02 %, and 96.58 % respectively, indicating a strong fit of the generated model to the data. A negative R squared value would suggest that the cumulative mean is a better predictor of response than the current model. In order to assess the precision of the model, the adequacy is gauged by the signal-to-noise ratio. An acceptable ratio is considered to be > 4. The signal-to-noise ratios for the present model were determined as 9.643 for Cd, 8.125 for Hg, and 12.965 for Pb, all of which indicate a substantial signal presence. All the statistical outcomes of the model collectively highlight its high accuracy and broad applicability as a second-order model. It effectively describes the responses observed in the experiments and ensures reliability in the results achieved.

**Fig. 2:**
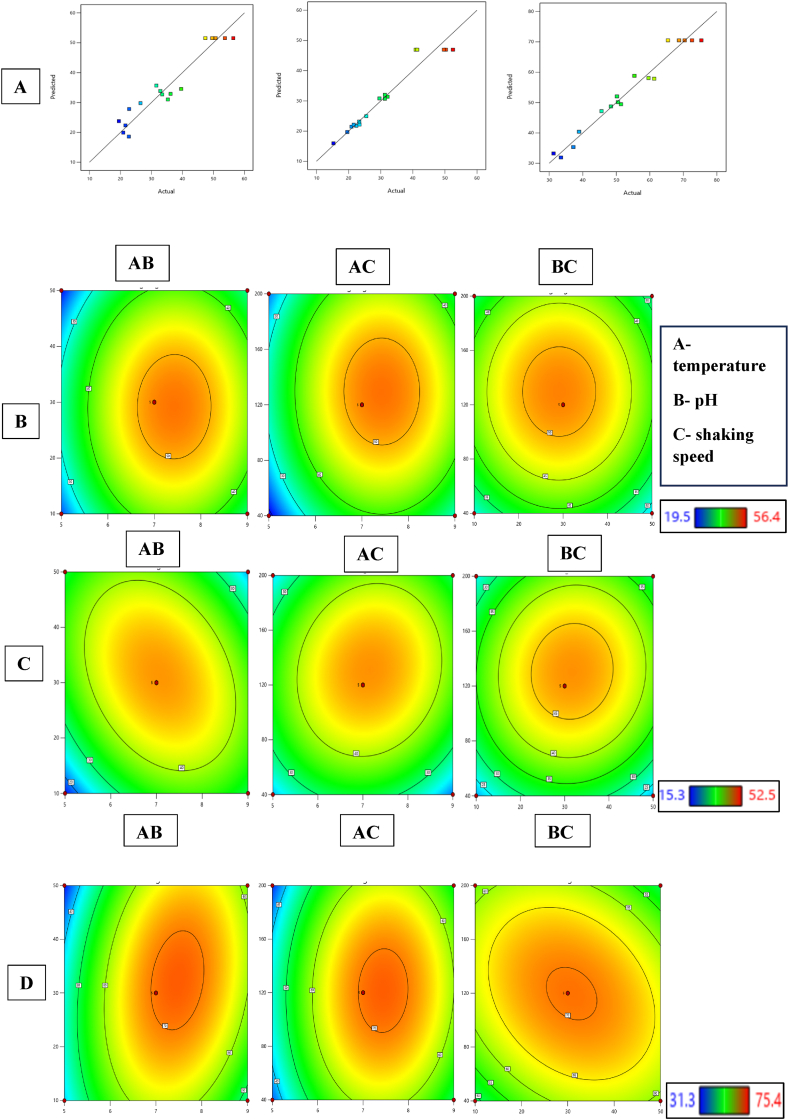
Optimization studies using Box-Behnken Design matrix **(A)** Normal probability plot of residuals, **(B)** contour plots illustrating the anticipated removal patterns of cadmium, **(C)** mercury, and **(D)** lead.

Fig. 2A displays the outcome of the response optimizer, employing a desirability function to illustrate the levels of independent variables that yield the most favorable predicted response. The plot of residuals adheres to the normal distribution principle when it exhibits a linear pattern [48]. Contour plots, generated through model regression, were created using Design Expert software. Fig. 2B-D presents contour plots depicting the predicted removal patterns of Cd, Hg, and Pb, as projected by equations (4), (5), (6) within the model. Each of these contour plots illustrates the impact of two factors while keeping the other variables at their higher levels (level 1). These graphical representations demonstrate the potential to achieve optimal yields exceeding 56 % for Cd, 52 % for Hg, and 75 % for Pb removal. In Fig. 2B, the contour plot depicts the relationship between pH and temperature. An increase in pH to 7 and a temperature rise to 30 °C notably enhanced the removal percentage of Cd, Hg and Pb by SMA3. The study revealed that elevating the temperature from 30 to 40 °C reduced the response rate, indicating an inhibition of SMA3 growth beyond the specified temperature range. Conversely, pH changes had an immediate impact on the removal rate. The removal rate exceeded at pH 7, while a subsequent decrease in pH led to a significant decline in removal of heavy metals. This suggests that a pH of 7 is ideal for the removal of heavy metals.

Fig. 2C displays the response curve for pH and shaking speed (rpm). Changes in shaking speed had a more pronounced effect on bacterial biomass and its efficiency in removing heavy metals. A decrease in removal percentage was observed when the shaking speed approached 80 rpm, but the removal of heavy metals increased with further increments to 120 rpm. Therefore, it can be inferred that the bacterial strain achieved the most effective heavy metals removal at an agitation velocity of 120 rpm. The heavy metal removal rate increased with 120 rpm at 30 °C, as demonstrated by the contour plot of shaking speed and temperature (Fig. 2D).

The model-based ideal operational conditions were identified to achieve the highest removal rates. Under the most favorable experimental conditions, where bacterial strain was introduced at a temperature of 30 °C, pH level of 7, and a shaking speed of 120 rpm, the analysis of responses showed the greatest removal percentages. Specifically, Cd, Hg, and Pb exhibited removal percentages of 69.5 %, 58.6 %, and 85.1 %, respectively. By juxtaposing single-parameter modifications with the combined influence of process parameters, it became evident that the combined effect led to an enhancement in the removal rate of heavy metal. Consequently, a strong concurrence was observed between the experimentally determined responses and the optimal responses predicted by the current model. In light of these findings, it can be affirmed that BBD has proven to be a highly effective approach for refining the parameters associated with removal of heavy metal, thus demonstrating its proficiency in this context.

### 3.6 Removal assay

**Fig. 3:**
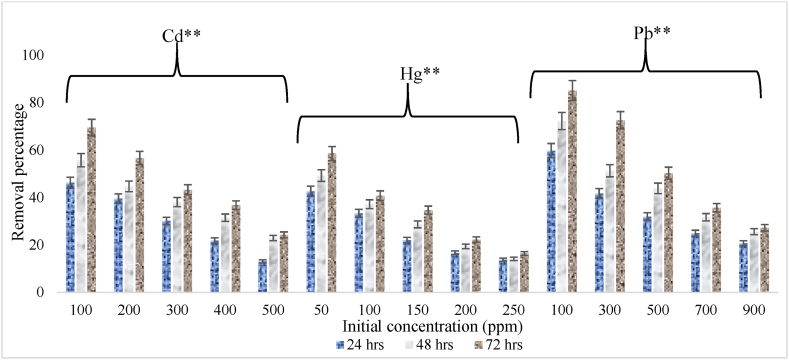
Removal percentage of heavy metal (cadmium, mercury, and lead) achieved by SMA3 at varied initial concentration under optimal conditions. Values are mean ± SD of triplicate sets. **Significant at 5 % according to ANOVA.

The effectiveness of the selected bacterial strain in the removal of Cd, Hg and Pb was investigated. Fig. 3 presents the removal rates observed at different concentrations after 24, 48, and 72 h. *Brevundimonas vancanneytii* SMA3 demonstrated a significant capability for removing Pb, followed by Cd and Hg from the medium. Specifically, the removal rate of Cd was 69.5 %, 55.8 %, and 46.3 % when the initial Cd concentration was 100 ppm, over 72, 48, and 24 h respectively. For Hg, the strain reduced concentrations by 58.6 %, 40.8 %, 34.7 %, 22.3 %, and 16.4 % from media with initial Hg concentrations of 50, 100, 150, 200, and 250 ppm, respectively, within 72 h. When it comes to Pb, the removal rate was 59.8 %, 72.3 %, and 85.1 % within 24, 48, and 72 h respectively, at an initial concentration of 100 ppm. *Gemella* sp. and *Micrococcus* sp. exhibited significant Pb removal, with respective values of 55.16 ± 0.06 % and 36.55 ± 0.01 %. These bacteria also demonstrated Cd removal, with removal rates of 50.99 ± 0.01 % and 38.64 ± 0.06 % respectively [31]. The bacterial strains displayed strong capabilities in removing heavy metals at lower concentrations, yet their effectiveness was limited when dealing with higher concentrations of heavy metals. This pattern was aligned with the findings of Kumar and Oommen [49], who observed a decline in removal percentages as the initial concentrations of Cd, Cr, Hg, and Pb increased, attributed to saturation of sorption sites on adsorbents.

### 3.7 SMA3 growth behavior under the influence of different substrates

The addition of nutrients has proven to be a highly effective method for accelerating the biodegradation process by enhancing the activity of microorganisms [50]. The strain SMA3 displayed robust growth in LB medium at 30 ± 2 °C during the exponential phase. The bacterial growth pattern was further explored in the presence of various carbon (starch, glycerol, glucose) and nitrogen (NaNO_3_, peptone, urea) substrates. The addition of carbon and nitrogen substrates to LB medium in the presence of Cd, Hg, and Pb led to a substantial enhancement in the growth rate of the bacterial isolate when compared to the control. Among the carbon sources, starch appeared to promote the most rapid growth, followed by glycerol and glucose. In terms of nitrogen sources, the bacterial strain demonstrated the fastest development in the presence of NaNO_3_, followed by peptone and urea (Table 5). Similar to this study, Ndayambaje et al. [51] observed that the presence of NaNO_3_ significantly augmented the growth rate of *Botryococcus* sp. Additionally, this presence led to a notable enhancement in the removal efficiency of Cd and Pb, with an increase of 10.5 % and 18.6 %, respectively.

**Table 5:** Effect of supplementary carbon and nitrogen sources on microbial activity after a 72-h period. Values are mean ± SD of triplicate sets.

	LB medium	Carbon substrate			Nitrogen Substrate		
	Control	Starch	Glycerol	Glucose	NaNO_3_	Urea	Peptone
OD 600 nm	0.586 ± 0.01	0.981 ± 0.02	0.671 ± 0.02	0.931 ± 0.04	0.712 ± 0.04	0.475 ± 0.06	0.623 ± .0.03

### 3.8 FTIR spectral analysis

**Fig. 4:**
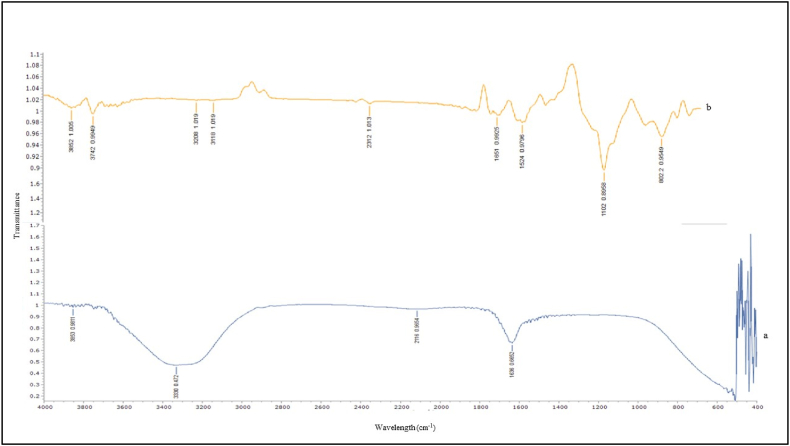
Comparative FTIR spectroscopy analysis of SMA3: **(a)** prior to the capture of heavy metal ions, and **(b)** subsequent to the capture of heavy metal ions.

Fig. 4 displays the FTIR spectra within the 4000-400 cm^−1^ band of the initial and heavy metals-loaded SMA3. The stretching vibrations of the C]O, C ^ C, and *C*–H groups are represented as peaks in the control spectrum at 1636, 2116, and 3330 cm^−1^, respectively. The spectrum of the sample devoid of heavy metals exhibited numerous absorption peaks, underscoring the intricate nature of the biomass being studied. After the absorption of Cd, Hg and Pb onto SMA3, the stretching vibrations of the groups *C*–*O*–C, C]O, *C*–H, and O–H were represented by the absorption peaks in the ranges of 1100–1200, 1500–1700, 3000–3200, and 3200–3400 cm^−1^, respectively. Changes in both wave number and intensity were distinctly observed in several absorption peaks subsequent to the capture of heavy metals, signifying that the mentioned functional groups played a primary role in the adsorption of heavy metals onto SMA3. Moreover, the asymmetric and symmetric bending vibrations of *C*–H correlated to the absorption peaks at 2312 cm^−1^. The changes implied that surface of SMA3 had undergone chemical reactions involving the heavy metal and the *C*–*O*–C, C]O, *C*–H, and O–H groups. In conclusion, the FTIR spectrum of the SMA3 that had been freed of heavy metals revealed the presence of hydroxyl, carboxyl, and amine groups, all of which were crucial in the removal of the heavy metal. Furthermore, the shifted peak intensity indicated that the functional group reduced electron densities and also altered the frequency and strength of their vibrations. Moreover, the peak in the 800-1000 cm^−1^ range may have been the consequence of carboxylic group ion exchange. Similar to this study, Quan et al. [42] reported that the biosorption process of heavy metal involve hydroxyl, carboxyl and amine functional groups. Microorganisms employ various mechanisms to mitigate the toxicity of heavy metals, such as forming bonds between their cell surfaces and metal ions [52,53]. The composition of bacterial cell surfaces, including carboxyl, phosphate, hydroxyl, and sulfate groups, plays a significant role in binding with these metals, ultimately leading to the deposition of metals on the cell surface or their accumulation in the intercellular space [54,55].

### 3.9 FESEM- EDX analysis

**Fig. 5:**
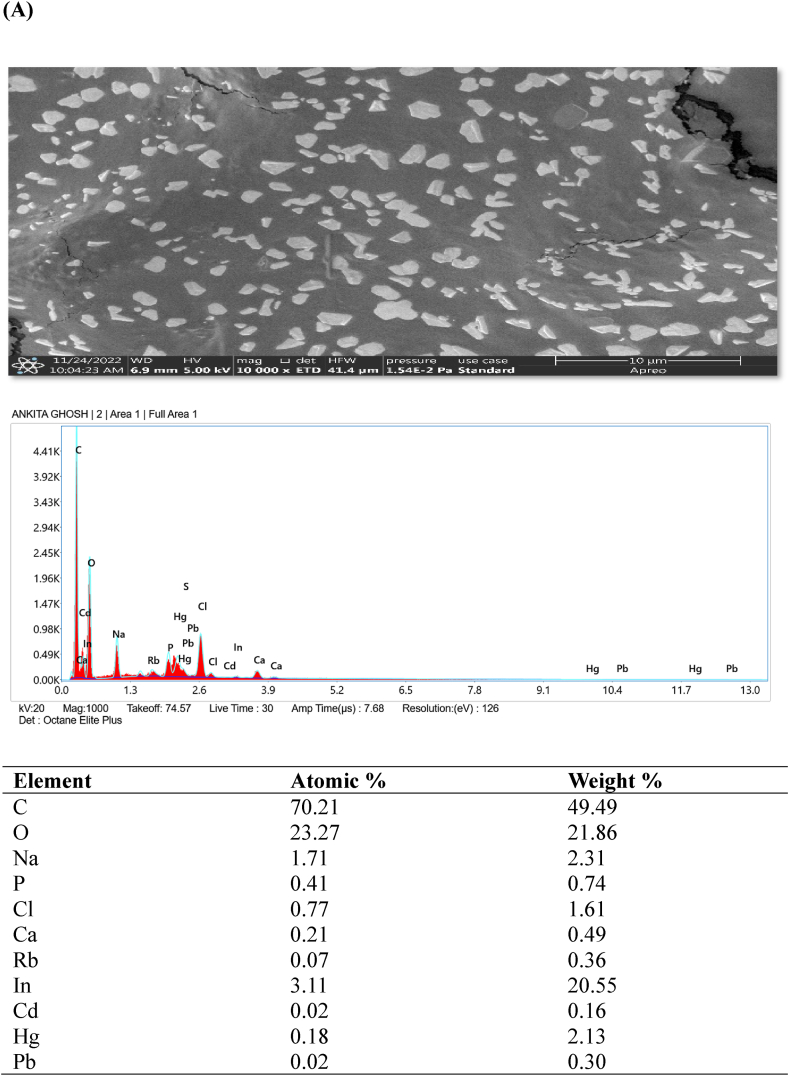

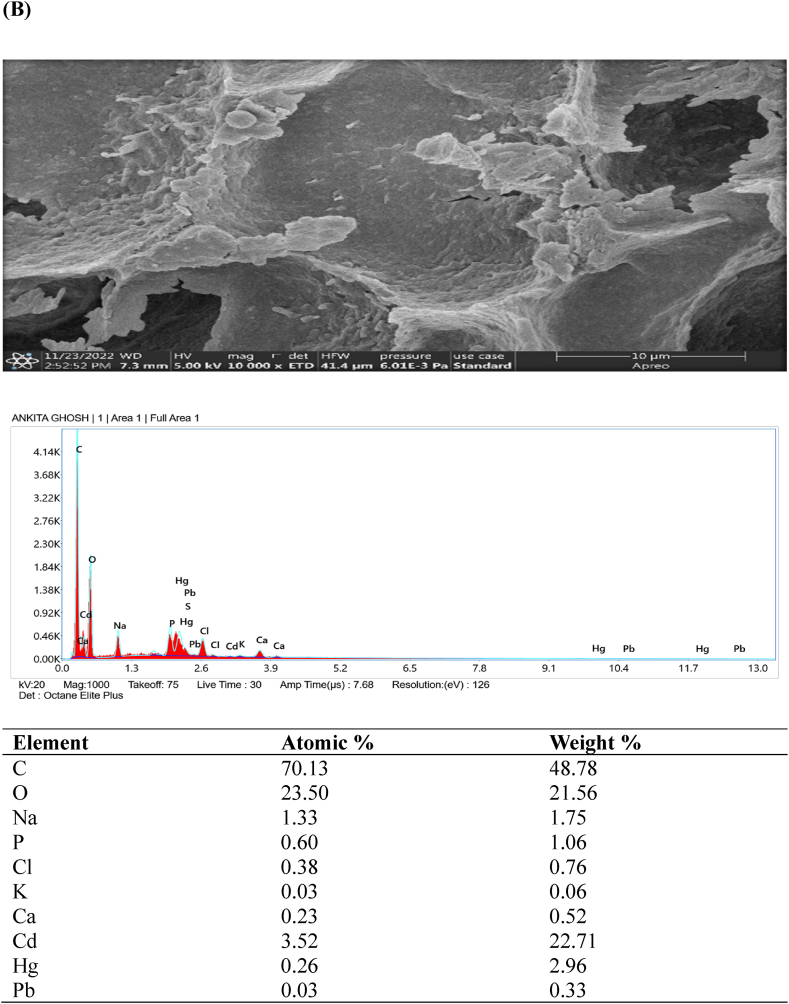

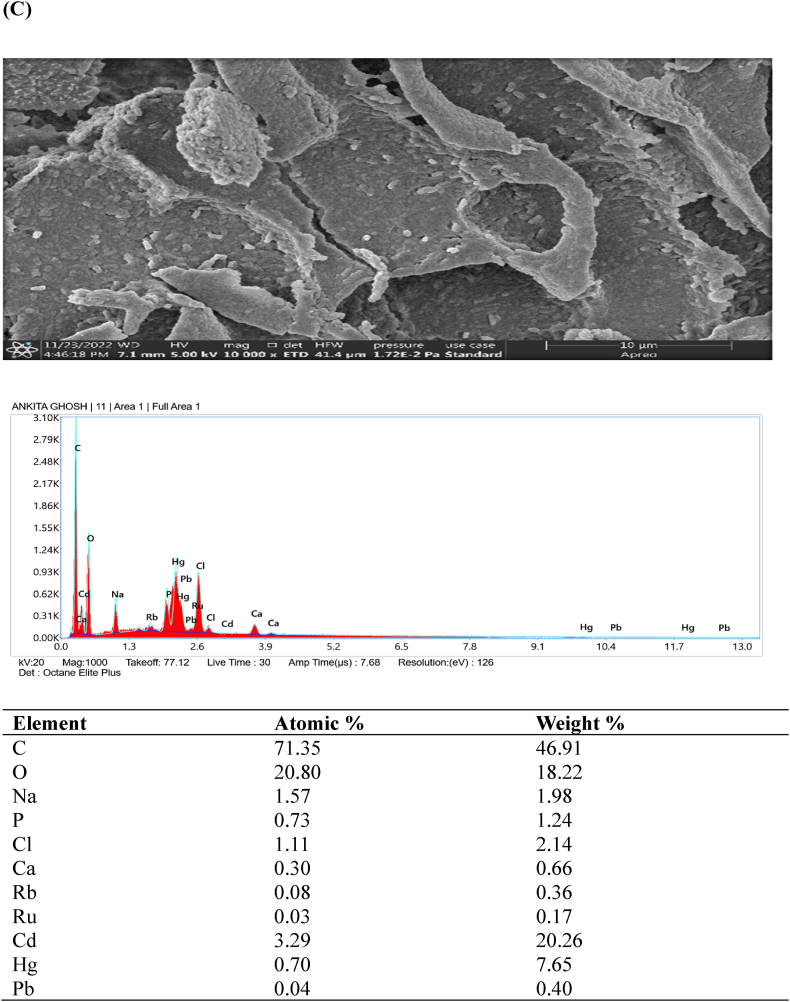

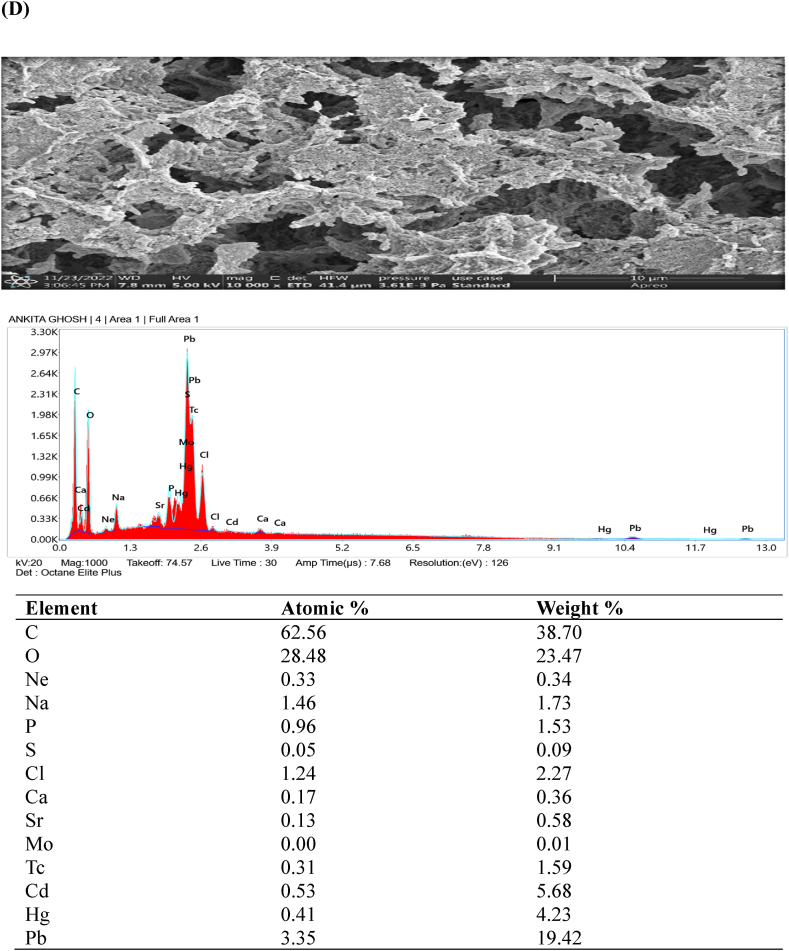
Examination of the morphology and energy spectrum: **(A)** prior to the adsorption of heavy metal ions, and **(B)** subsequent to the adsorption of cadmium, **(C)** mercury, and **(D)** lead ions.

Examination of the original and heavy metal-loaded SMA3 samples was conducted using field emission scanning electron microscopy (FESEM) to delve deeper into the metal removal process. In Fig. 5A–D, an energy spectrum analysis highlights the distinctive components and morphology of SMA3 both prior to and after capturing heavy metals (Cd, Hg, and Pb). FESEM observations unveiled that the surface of bacterial cells exhibited a smooth and even texture prior to the removal of heavy metals (Fig. 5A). Similar study showed that magnification range spanning from 5000 × to 10,000 × was employed, coupled with a 20 kV acceleration voltage. The FESEM micrographs provided clear and visually striking evidence of the distinct morphological disparities between the bacterial biomass in its natural state (control) and when subjected to heavy metal exposure. In the case of control, the surface morphology was notably characterized by extreme fineness, remarkable smoothness, and a high degree of homogeneity [56]. However, subsequent to the adsorption of heavy metals onto the bacterial cell surfaces, the texture became uneven and rugged (Fig. 5B–D). Though, the capacity for adsorption was somewhat constrained, and only a fraction of bacterial cells exhibited observable morphological changes. The presence of heavy metals on the bacterial cell surface implies the potential involvement of these cells in the removal of heavy metals through adsorption [57]. The capturing of heavy metals by SMA3 is attributed to the presence of various functional groups such as hydroxyl, amino, and carboxyl groups. This capacity stems from the diverse structural elements inherent in SMA3.

The detoxification and adsorption of heavy metals by bacteria likely involve multiple mechanisms, including cell surface adsorption, organic acid coordination reactions, and biological flocculation processes, all occurring simultaneously [58]. The cell surface is equipped with ions that can effectively interact with heavy metal ions, facilitating the adsorption and immobilization of these metals [59]. For example, during the adsorption of Cd (II) by *Bacillus cereus*, the release of K^+^, Ca^2+^, Na^+^, and Mg^2+^ was observed, indicating an ion exchange mechanism that facilitated this process [60]. The predominant mechanism underlying bacterial surface adsorption is ion exchange. In the context of this study, the bacteria likely contributed to the removal of heavy metal ions by forming complexes and engaging in ion exchange, effectively immobilizing the heavy metal ions on the bacterial surface.

### 3.10 Phytotoxicity assay

**Fig. 6:**
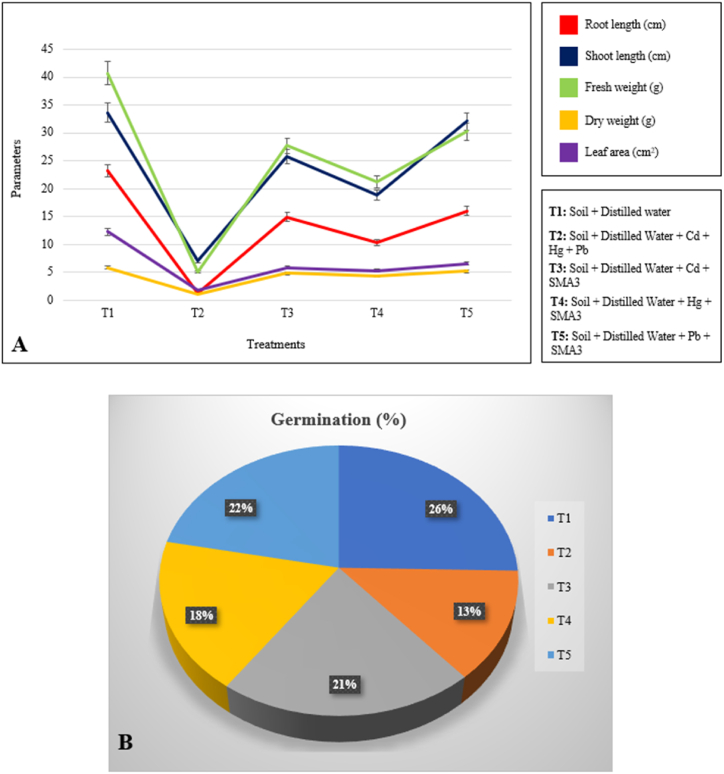
(A) Variability in growth parameters of *Solanum lycopersicum* in the presence or absence of the potent bacterial strain **(B)** Germination percentage exhibited by the different treatments.

To analyze the effect of bacterial strain SMA3 on the removal efficiency of heavy metals in the contaminated soil along with phytotoxicity effect; germination percentage, shoot and root length, fresh and dry weight along with leaf area of *Solanum lycopersicum* plants was studied. In this study, it was explored that highest growth parameter was found in control followed by SMA3 treated soil and the lowest in contaminated soil as depicted in Fig. 6A. In case of germination percentage, Fig. 6B shows that the highest value was observed in control (82.6 %) and lowest in the contaminated soil (43.4 %), however germination percentage improves when treated with the selected inoculum. This study showed that in heavy metals contaminated soil, sprouting of seeds and seedling development are hindered, but SMA3 treatment lowered the heavy metal toxicity in soil as illustrated by the improved plant growth parameters. According to the findings, heavy metal reduced the germination percentage and growth parameters of the plant. Overall, the study indicated the importance of investigating the negative effects of heavy metals on tomato plants, a good source of essential nutrients such as vitamins (like vitamin C, vitamin A, and vitamin K), minerals (like potassium), and dietary fiber [45].

## 4 Conclusion

A cost efficient and environment friendly bio remedial process for reducing heavy metal ions was fabricated employing biocatalytic potential of a native bacterial strain *Brevundimonas vancanneytii* SMA3. The current study has characterized SMA3 as a highly potent multi-metal reducing strain, achieving impressive removal percentages: 69.5 % for Cd, 58.6 % for Hg, and 85.1 % for Pb, all within a short 72-h period. Employing response surface methodology (RSM) further optimized the removal process, enhancing its effectiveness. The reduction in the absorption of distinct peaks corresponding to heavy metals observed in FTIR spectra provided confirming evidence of the removal process. This confirmation was reinforced by examining the microstructural characteristics of the bacterial biomass fraction using Field Emission Scanning Electron Microscopy (FESEM). Furthermore, the study delved into the practical application of this bioremediation strategy by conducting phytotoxicity assays. These assays revealed that soil treated with SMA3 exhibited improved growth parameters and germination percentages compared to untreated soil.

## Data availability statement

The data that has been used is confidential.

## CRediT authorship contribution statement

**Ankita Ghosh:** Writing – review & editing, Writing – original draft, Visualization, Validation, Software, Resources, Project administration, Methodology, Investigation, Funding acquisition, Data curation, Conceptualization. **Diksha Sah:** Writing – review & editing. **Moumita Chakraborty:** Writing – review & editing. **J.P.N. Rai:** Supervision, Formal analysis.

## Declaration of competing interest

The authors ensure that this research article does not have any conflict of interest.
