# Supplementary material for: Bio-mediated detoxification of heavy metal contaminated soil and phytotoxicity reduction using novel strain of Brevundimonas vancanneytii SMA3
Source: Heliyon. 2023 Nov 17;9(11):e22344. doi: 10.1016/j.heliyon.2023.e22344 (PMC10696005; doi:10.1016/j.heliyon.2023.e22344)
Supplement: Multimedia component 1 [file mmc1.docx]

**Supplement 1:**

Dose- dependent MIC of the selected isolate SMA3

| Concentration (ppm) | Cadmium (Cd) | Mercury (Hg) | Lead (Pb) |
| --- | --- | --- | --- |
| 100 | +++ | +++ | +++ |
| 200 | +++ | +++ | +++ |
| 300 | +++ | +++ | +++ |
| 400 | +++ | +++ | +++ |
| 500 | +++ | ++ | +++ |
| 600 | +++ | ++ | +++ |
| 650 | +++ | + | +++ |
| 700 | +++ | - | +++ |
| 750 | +++ | - | +++ |
| 800 | ++ | - | +++ |
| 850 | ++ | - | +++ |
| 900 | - | - | ++ |
| 950 | - | - | ++ |
| 1000 | - | - | ++ |
| 1050 | - | - | ++ |
| 1100 | - | - | + |
| 1120 | - | - | + |
| 1150 | - | - |  |

Degree of growth on NA plates, +++ Good, ++ Moderate, + Survival, - No growth

**Supplement 2A:**

ANOVA for quadratic model of cadmium removal using bacterial strain SMA3

| **Source** | **Sum of Squares** | **Df** | **Mean Square** | **F- value** | **p- value** |  |
| --- | --- | --- | --- | --- | --- | --- |
| **Model** | 2255.38 | 9 | 250.60 | 8.97 | 0.0043 | significant |
| 1. pH | 295.25 | 1 | 295.25 | 10.57 | 0.0140 |  |
| 1. Temperature | 136.48 | 1 | 136.48 | 23.20 | 0.0644 |  |
| 1. Shaking speed | 148.02 | 1 | 148.02 | 11.27 | 0.0231 |  |
| AB | 142.25 | 1 | 142.25 | 15.12 | 0.0095 |  |
| AC | 106.25 | 1 | 106.25 | 22.11 | 0.0063 |  |
| BC | 102.25 | 1 | 102.25 | 8.13 | 0.0082 |  |
| A^2^ | 845.13 | 1 | 845.13 | 20.25 | 0.0009 |  |
| B^2^ | 435.28 | 1 | 435.28 | 15.58 | 0.0056 |  |
| C^2^ | 431.01 | 1 | 431.01 | 15.43 | 0.0057 |  |
| **Residual** | 195.54 | 7 | 27.93 |  |  |  |
| Lack of Fit | 145.65 | 3 | 48.55 | 3.89 | 0.1111 | not significant |
| Pure Error | 49.89 | 4 | 12.47 |  |  |  |
| **Cor Total** | 2450.92 | 16 |  |  |  |  |

**Supplement 2B:**

ANOVA for quadratic model of mercury removal using bacterial strain SMA3

| **Source** | **Sum of Squares** | **Df** | **Mean Square** | **F- value** | **p- value** |  |
| --- | --- | --- | --- | --- | --- | --- |
| **Model** | 2057.98 | 9 | 228.66 | 13.01 | 0.0014 | significant |
| 1. pH | 131.52 | 1 | 131.52 | 0.7690 | 0.0409 |  |
| 1. Temperature | 202.48 | 1 | 202.48 | 1.16 | 0.0316 |  |
| 1. Shaking speed | 173.21 | 1 | 173.21 | 4.16 | 0.0806 |  |
| AB | 165.12 | 1 | 165.12 | 9.39 | 0.0182 |  |
| AC | 125.25 | 1 | 125.25 | 1.45 | 0.0676 |  |
| BC | 166.50 | 1 | 166.50 | 0.3699 | 0.0562 |  |
| A^2^ | 534.55 | 1 | 534.55 | 30.41 | 0.0009 |  |
| B^2^ | 456.94 | 1 | 456.94 | 25.99 | 0.0014 |  |
| C^2^ | 578.10 | 1 | 578.10 | 32.88 | 0.0007 |  |
| **Residual** | 123.06 | 7 | 17.58 |  |  |  |
| Lack of Fit | 6.07 | 3 | 2.02 | 0.0692 | 0.9734 | not significant |
| Pure Error | 116.99 | 4 | 29.25 | 29.25 |  |  |
| **Cor Total** | 2181.04 | 16 |  |  |  |  |

**Supplement 2C:**

ANOVA for quadratic model of lead removal using bacterial strain SMA3

| **Source** | **Sum of Squares** | **Df** | **Mean Square** | **F- value** | **p- value** |  |
| --- | --- | --- | --- | --- | --- | --- |
| **Model** | 2981.00 | 9 | 331.22 | 22.00 | 0.0002 | significant |
| 1. pH | 542.85 | 1 | 542.85 | 16.05 | 0.0005 |  |
| 1. Temperature | 288.13 | 1 | 288.13 | 9.12 | 0.0067 |  |
| 1. Shaking speed | 101.32 | 1 | 101.32 | 6.70 | 0.0093 |  |
| AB | 94.09 | 1 | 94.09 | 6.25 | 0.0410 |  |
| AC | 155.26 | 1 | 155.26 | 13.66 | 0.0056 |  |
| BC | 147.82 | 1 | 147.82 | 15.26 | 0.0055 |  |
| A^2^ | 179.21 | 1 | 179.21 | 13.25 | 0.0001 |  |
| B^2^ | 228.24 | 1 | 228.24 | 15.16 | 0.0060 |  |
| C^2^ | 359.29 | 1 | 359.29 | 23.86 | 0.0018 |  |
| **Residual** | 105.41 | 7 | 15.06 |  |  |  |
| Lack of Fit | 47.73 | 3 | 15.91 | 1.10 | 0.4452 | not significant |
| Pure Error | 57.68 | 4 | 14.42 |  |  |  |
| **Cor Total** | 3086.42 | 16 |  |  |  |  |
